# Diffuse Alveolar Hemorrhage in an Undiagnosed Systemic Lupus Erythematosus Patient

**DOI:** 10.7759/cureus.38421

**Published:** 2023-05-02

**Authors:** Qais M Salah, Maram Albandak, Mohammed Ayyad, Dana Sayyed Ahmad, Saed I Atawnah

**Affiliations:** 1 Internal Medicine, Al-Quds University, Jerusalem, PSE; 2 Internal Medicine, Al-Ahli Hospital, Hebron, PSE

**Keywords:** autoimmune disease, rheumatologic disorders, thrombocytopenia, systemic lupus erythromatosus, diffuse alveolar hemorrhage

## Abstract

Systemic lupus erythematosus (SLE) is a chronic multisystem autoimmune disease characterized by a wide range of clinical manifestations affecting multiple organs. While standardized diagnostic criteria are commonly used, the lack of pathognomonic presenting signs and symptoms often makes the diagnosis challenging. Of the many pulmonary manifestations, diffuse alveolar hemorrhage (DAH) is one of the most severe complications caused by the disruption of the capillary alveolar interface. Although this condition is rarely encountered, it has a rapidly progressive course and can be life-threatening, which warrants a prompt diagnostic workup and an aggressive therapeutic approach.

We report a case of a 58-year-old female who presented to the emergency department with dyspnea and multiple episodes of hemoptysis. Further investigations revealed anemia, thrombocytopenia, and diffuse bilateral infiltrates on high-resolution computed tomography, consistent with DAH in a patient with undiagnosed SLE.

## Introduction

Systemic lupus erythematosus (SLE) is an autoimmune disease characterized by a broad spectrum of clinical presentations ranging from mild cutaneous and laboratory manifestations to severe end-organ damage [1]. One of the rare and potentially fatal initial presentations of SLE is diffuse alveolar hemorrhage (DAH), which occurs in less than 5% of cases as an initial presenting symptom [2]. DAH is a form of diffuse pulmonary bleeding that specifically refers to bleeding that is limited to the alveolar area [3]. It is a rare life-threatening complication of SLE with a high mortality rate reaching up to 50% [2]. Thus, a high index of clinical suspicion is essential to promptly diagnose and treat this condition. The constellation of hemoptysis, anemia, and diffuse radiographic pulmonary infiltrates comprise the classic presentation of DAH. However, nearly one-third of cases present without hemoptysis, or with nonspecific findings on radiological studies, leading to misdiagnosis [4].

We herein describe a case of DAH as the initial presenting symptom in an undiagnosed SLE patient, manifesting as hemoptysis, anemia, and diffuse bilateral pulmonary infiltrates.

## Case presentation

**Figure 1:**
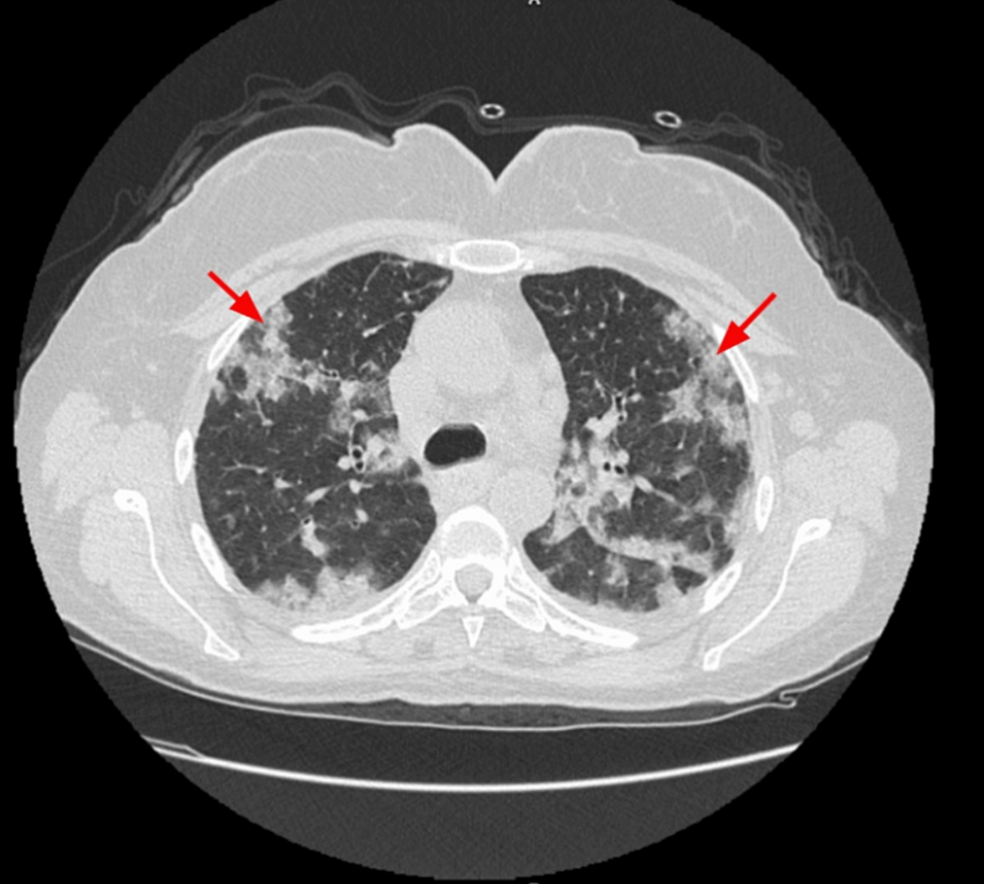
HRCT of the chest HRCT of the chest showing wide areas of patchy infiltrates in multiple areas within the lung parenchyma (red arrows). These findings alongside the patient’s presentation are suggestive of hemorrhage in the lungs.

**Figure 2:**
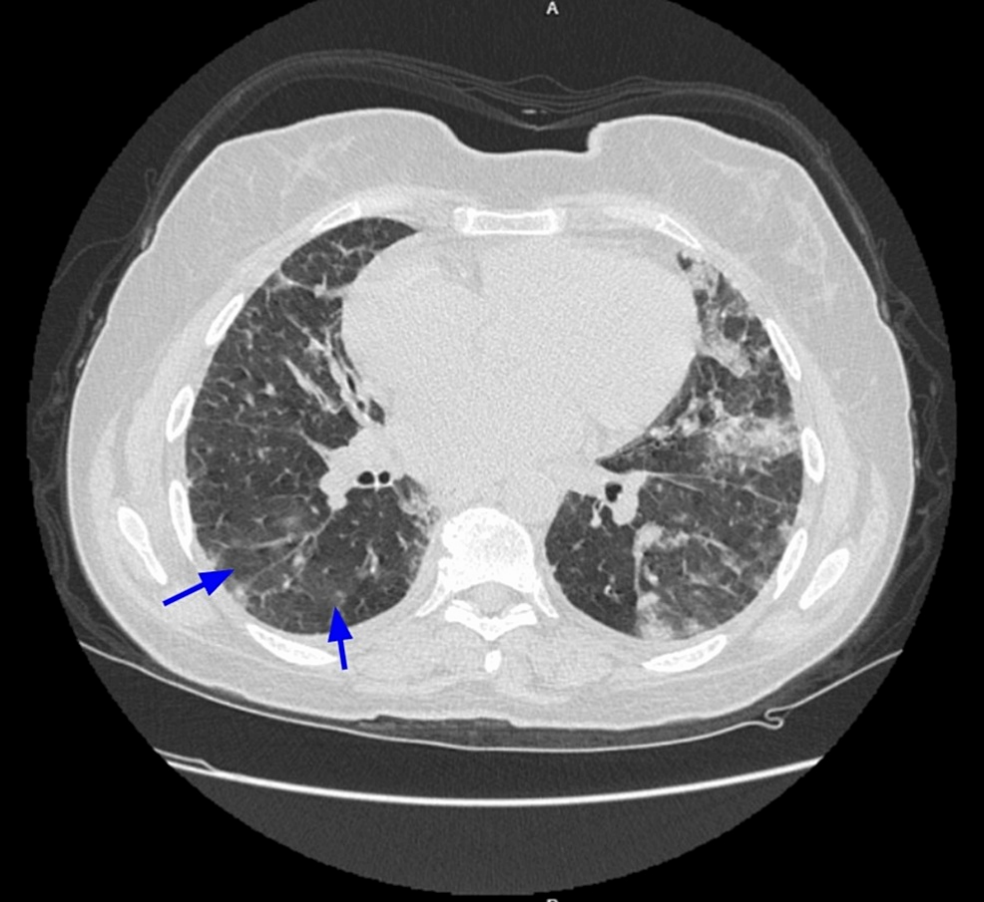
HRCT of the chest HRCT of the chest showing ground-glass opacities in the lung parenchyma (blue arrows).

A 58-year-old female nonsmoker patient presented to the ED complaining of a sudden onset of shortness of breath that was severe enough to awaken her from sleep, followed by multiple episodes of hemoptysis. Upon questioning, the patient reported she had heavy gum bleeding after a dental procedure just four days ago. She also stated that she experienced arthralgia for the past month associated with multiple episodes of headache, chills, and enlarged cervical lymphadenopathy. Additionally, a bilateral lower limb petechial rash was noticed with scarring alopecia on her head. On admission, her physical examination was notable for diffuse bilateral crackles on her chest auscultation. Vital signs revealed a temperature of 36.8 °C, blood pressure of 102/76 mmHg, heart rate of 143 beats per minute, respiratory rate of 34 breaths per minute, and an oxygen saturation of 75% on room air. Subsequently, a chest radiograph was ordered and showed bilateral diffuse infiltrates. A high-resolution computed tomography (HRCT) of the lungs revealed bilateral patchy infiltrates (Figure 1), as well as widespread areas of ground glass and consolidative opacities involving 50-70% of the lung parenchyma, which were highly suggestive of DAH (Figure 2).

Laboratory investigations revealed a hemoglobin of 8.7 g/dL (normal range in females: 12-15 g/dL), as well as a platelet count of 3.9x10^3^/μL (normal range: 150-400 x10^3^/μL) and white blood cell count of 7x10^3^/μL (normal range: 5-10 x10^3^/μL). Furthermore, a nasal swab for COVID-19 was negative. Arterial blood gas showed evidence of severe respiratory alkalosis, with a pH of 7.75 (normal range: 7.35-7.45), partial pressure of carbon dioxide of 11.1 mmHg (normal range: 35.0-48.0 mmHg), partial pressure of oxygen of 130 mmHg (normal range: 83-108 mmHg), and bicarbonate (HCO3-) of 16 mEq/L (normal range: 23-28 mEq/L).

The patient’s clinical and historical presentation raised suspicion about a rheumatological disease. Thus, after the initial stabilization, a thorough evaluation for rheumatologic diseases revealed positive antinuclear antibodies, rheumatoid factor, and anti-double-stranded DNA (anti-dsDNA) antibodies. Moreover, the erythrocyte sedimentation rate was markedly elevated. Serum cytoplasmic antineutrophil cytoplasmic autoantibodies, perinuclear anti-neutrophil cytoplasmic antibodies, anticardiolipin antibodies, antithrombin antibodies, antiphospholipid antibodies, and lupus anticoagulant antibodies were all negative. Serum complements C3 and C4 were within normal levels (Table 1).

**Table 1:** Comprehensive laboratory tests of our patient on admission anti-dsDNA: anti-double-stranded DNA

Laboratory Test	Result
Hemoglobin (g/dL)	8.7 g/dL
Platelets count (x 10^3^/μL)	3.9x10^3^/μL
White blood cells count (x 10^3^/μL)	7x10^3^/μL
pH	7.75
Partial pressure of carbon dioxide (mmHg)	11.1 mmHg
Antinuclear antibody	Positive
Anti-dsDNA antibodies	Positive
Rheumatoid factor	Positive
Antiphospholipid antibodies	Negative
Lupus anticoagulant antibodies	Negative
Serum complements C3 and C4	Normal

**Figure 3:**
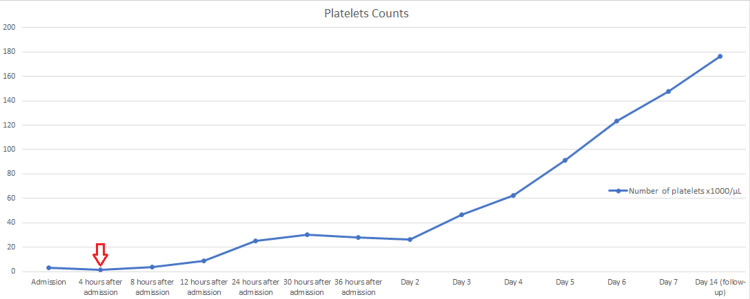
A diagram showing the trend of platelet counts in our patient during her hospital stay and post-discharge The red arrow indicates the beginning of steroid therapy administration.

Ultimately, the patient was diagnosed with SLE and received supportive care. She was urgently transferred to the ICU where she was given six units of platelets. Pulse steroid therapy with methylprednisolone 1,000 mg was initiated to manage her severe presentation and continued for three days, along with high-dose intravenous cyclophosphamide. The patient’s platelet counts gradually improved over the course of her hospital stay, as shown in the diagram below (Figure 3).

**Figure 4:**
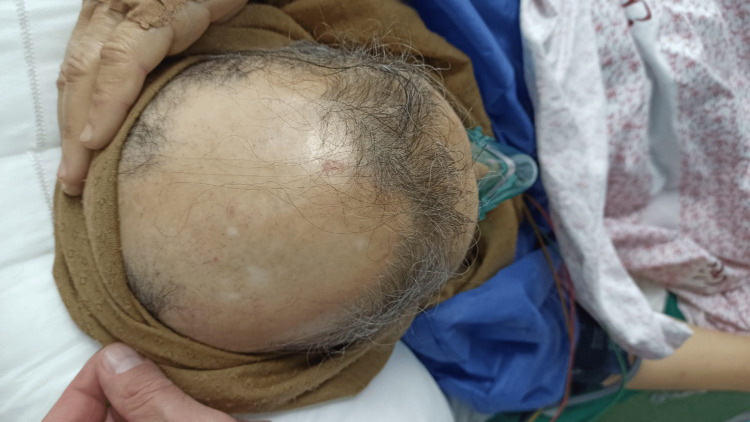
Scarring alopecia on the patient's scalp

**Figure 5:**
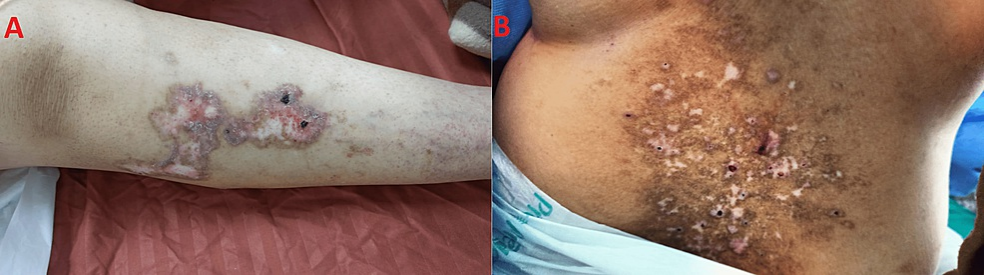
Scaling rash on the forearm and the back consistent with discoid lupus (A) Skin rash on the left forearm. (B) Skin rash on the back.

After the patient was stabilized, further medical history revealed heart failure with an ejection fraction of 35% diagnosed four years ago. Additionally, the patient reported hair thinning seven years ago, which progressed to scarring alopecia (Figure 4), as well as a skin rash that started 20 years ago diagnosed as eczematous dermatitis based on a previous skin biopsy (Figure 5).

Once the patient’s condition stabilized, a repeat skin biopsy revealed discoid lupus erythematosus. Her family history was positive for rheumatoid arthritis in her aunt.

The patient's overall condition improved three days later. She was transferred to the medical ward for further management of her underlying condition and discharged one week later. On follow-up, the patient reported no complaints, improved general health, her complete blood count (CBC) was within normal range, and she was prescribed hydroxychloroquine as a mainstay therapy for her newly diagnosed SLE.

## Discussion

SLE is an autoimmune disease characterized by various manifestations, including chronic fatigue, skin rash, arthritis, glomerulonephritis, and neurological, pulmonary, and cardiovascular involvement. Respiratory tract involvement is present in up to 50-70% of SLE cases and encompasses pleuritis, infiltrating pneumonia, bronchiolitis obliterans, pulmonary hypertension, antiphospholipid syndrome, and DAH. Defective phagocytosis, immune complexes formed by antibodies to nuclear and cytosolic antigens, autoantibodies, and complement depletion are responsible for these pulmonary manifestations. The exact etiology for DAH is yet undetermined, but it is presumed to be related to immune complexes inciting inflammation in the alveolar capillaries (pulmonary capillaritis), or non-inflammatory bland hemorrhage that damages the basement membrane, causing leakage of RBCs into the alveolar space [5]. Although the exact trigger of the capillaritis is not known, antiphospholipid antibodies have been reported as possible initiating factors in some cases of DAH [6,7].

The incidence of DAH in patients with SLE ranges from 0.6% to 5.4% with an average estimated mortality rate of 50%. However, focal and diffuse collections of RBCs were identified in 30-66% of autopsies performed on patients with SLE, which suggests the presence of unidentified cases and subclinical disease [5].

Kazzaz et al. described 22 cases of DAH in a large SLE cohort of approximately 1000 patients, which revealed a notable link between a history of thrombocytopenia and DAH, similar to our patient. This is of pertinent importance as dyspnea is a more common presentation than hemoptysis, which is only present in about one-in-four presentations [7]. Although patients with DAH present most frequently with dyspnea, cough, hemoptysis, and pleuritic chest pain are other possible presentations as well [6].

DAH is most prevalent in young women with a mean age of 27 years. It can present early after SLE diagnosis with an average of 35 months after the onset [5]. Moreover, lupus nephritis has been linked to DAH, with active renal disease reported in as many as 64-100% of patients with lupus DAH [7]. Conversely, our patient first presented with DAH at the age of 58 after having undiagnosed manifestations of SLE for 18 years. She presented with severe dyspnea followed by hemoptysis, and CBC on admission revealed thrombocytopenia. Interestingly, she had no evidence of lupus nephritis by history and examination, and no evidence of proteinuria or hematuria on laboratory testing.

Although DAH complicates 2-4% of SLE cases, it should be considered in patients exhibiting signs and symptoms of pulmonary hemorrhage in the appropriate clinical context. The differential diagnosis for SLE-induced DAH is broad and includes small- and medium-vessel vasculitis, other connective tissue disorders such as rheumatoid arthritis and Sjogren’s syndrome, coagulation disorders, and drug-induced etiologies such as cytotoxic medications and the inhalation of crack cocaine, all of which should be investigated and excluded in this population. Additionally, SLE can lead to acute alveolar pneumonitis, which manifests with clinical and radiological features that are similar to DAH [8]. Therefore, it is critical to distinguish between these two conditions in individuals with SLE, as they require different therapeutic approaches.

The presence of the “classic triad” of hemoptysis, rapid decrease in hemoglobin, and the presence of new diffuse infiltrates on chest X-ray or HRCT should raise suspicion for DAH [9]. The diagnosis is made with a combination of history, physical examination, and laboratory evaluation, including sudden onset shortness of breath, drop in hemoglobin, elevated single-breath diffusing capacity for carbon monoxide, and the presence of pulmonary interstitial or alveolar infiltrates on imaging, especially in cases of active SLE with a Disease Activity Index over 10. Other factors associated with an increased risk of DAH include elevated serological titers of anti-dsDNA, thrombocytopenia, leucopenia, C3 hypocomplementemia, capillaritis, and the presence of immune complexes in biopsies [5].

High-dose glucocorticoids have formed the basis for DAH treatment, with notably increased survival rates in patients who received higher pulses of methylprednisolone (a total dose of 4-8 grams) compared to patients who received the conventional methylprednisolone treatment dose of 3 grams [10]. Methylprednisolone should be continued until the hemorrhage has stopped [5]. Cyclophosphamide and plasmapheresis are other frequently used therapies, commonly used in combination with increased survival rates [5]. A systematic review of DAH therapies by Ednalino et al. analyzing 140 patients with 174 episodes found that corticosteroids were the most frequent treatment modality used (98%), followed by cyclophosphamide (54%), plasmapheresis (31%), azathioprine (7%), intravenous immunoglobulin (5%), mycophenolate (3%), rituximab (6%), and stem cell transplantation [11].

The mortality of DAH has improved over time. Factors associated with an increased risk of mortality include acute fatal hemoptysis, a requirement for mechanical ventilation, infections, thrombocytopenia, elevated APACHE II score, and elevated creatinine [5,10].

## Conclusions

DAH is a rare potentially fatal complication of SLE that’s associated with a high rate of morbidity and mortality despite aggressive treatment. Interestingly, DAH can be the initial presentation of SLE and should warrant a comprehensive clinical, laboratory, and radiological investigation for the condition. The pathophysiology of SLE-induced DAH is suspected to involve a combination of coagulopathy, immune-complex deposition, and pulmonary capillaritis disrupting the alveolar-capillary interface. The treatment approach for DAH in patients with SLE is focused on managing the underlying autoimmune disorder. The use of immunosuppressants, immunomodulators, and stem cell transplantation has been found to be effective in producing a clinically significant response. More prospective randomized controlled trials are necessary to determine the optimal therapeutic guidelines for DAH.
